# First record of *Anopheles* (*Anopheles*) *hyrcanus* (Pallas 1771) (Diptera: Culicidae) in Poland

**DOI:** 10.1186/s13071-023-05974-z

**Published:** 2023-10-04

**Authors:** Renke Lühken, Norbert Becker, Dagmara Dyczko, Felix G. Sauer, Konstantin Kliemke, Jonas Schmidt-Chanasit, Katarzyna Rydzanicz

**Affiliations:** 1 Bernhard-Nocht-Institute for Tropical Medicine, Bernhard-Nocht-Str. 74, Hamburg, Germany; 2 Faculty of Biosciences, University of Heidelberg, Im Neuenheimer Feld 230, 69120 Heidelberg, Germany; 3 Institute of Dipterology (IfD)/KABS, Georg-Peter-Süß-Str. 3, 67346 Speyer, Germany; 4 Department of Microbial Ecology and Environmental Protection, University of Wroclaw, Przybyszewskiego Str. 63/77, 51-148 Wrocław, Poland; 5 Faculty of Mathematics, Informatics and Natural Sciences, Universität Hamburg, Hamburg, Germany; 6 Sycowska str. 12B/6, 51-319 Wrocław, Poland

**Keywords:** Exotic mosquitoes, *Anopheles hyrcanus*, Poland, First record

## Abstract

The spatial distribution of mosquito species in the course of globalization and climate warming is highly dynamic. Different studies have demonstrated the spread and establishment of thermophilic mosquito species, potentially increasing the prevalence of ‘nuisance’ mosquitoes and the local transmission of pathogens. Here we report the first recorded sampling of *Anopheles hyrcanus* in Wrocław, southwest Poland. This is the most northern detection of this species to date in Europe. Future spread and population development of this potential vector of malaria parasites, viruses or zoonotic helminths, such as *Dirofilaria* spp., must be monitored carefully. Potential factors underlying the spread of this species are discussed.

**Graphical Abstract:**

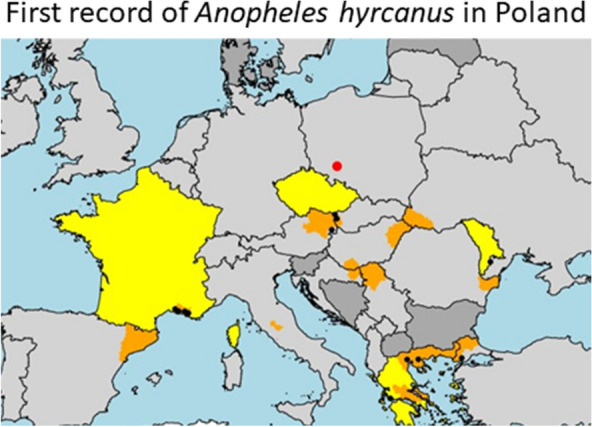

Mosquitoes (Diptera: Culicidae) are a main focus of medical entomological research. While several mosquito species are considered to be ‘nuisance’ mosquitoes only, others are vectors of various pathogens, such as malaria parasites or arboviruses [1]. The spread of exotic vector mosquitoes increases the threats to global health, not only in the tropics but also in temperate climate zones [2, 3]. Several *Aedes* mosquito species are particularly known for their high potential to colonize new regions far beyond their native geographical distribution. The most important trait enabling such long-distance dispersal is the high resistance of the eggs of many *Aedes* species to desiccation, i.e. the eggs can survive dryness for months or sometimes even years [4]. In addition, successful invasive mosquito species are well adapted to modern human environments in that they are able to colonize artificial breeding sites, such as used tires, and feed on humans [3]. The intercontinental spread of *Aedes* species is predominantly facilitated through the global transport of eggs with different commercial goods (e.g. used tires and plants such as lucky bamboo). Once locally established, medium-scale dispersal is mediated by the transport of adults as blind passengers in vehicles and boats [5]. In Europe, the best known example of an invasive mosquito species is the Asian tiger mosquito (*Aedes albopictus*) [3]. In the early 1990s, this species was introduced from the USA to Italy and then rapidly spread within Italy and to areas around the Mediterranean basin. Current observations have revealed the spread of this mosquito species towards Central Europe, with several populations reported to be established in areas north of the Alps [6].

For mosquito species which do not lay eggs that can remain dormant for an extended period of time (e.g. *Culex*, *Culiseta* or *Anopheles*), long-distance dispersal is less likely, but not impossible. For example, the *Culex coronator* complex, which was first described in Trinidad and Tobago, has shown a rapid range expansion throughout the southern US states [7]. The regular import of exotic taxa of the above-mentioned genera into Europe has also been reported [8]. At the same time, there are indications of changes in the distribution of mosquito species native to southern Europe, with a trend towards Central Europe, such as the spread of *Culiseta longiareolata* to Germany [9].

Monitoring programs aimed at assessing exotic mosquito populations can provide information on the local risk of nuisance mosquitoes or pathogen transmission. In addition, such programs allow the implementation of a quick response, such as specific control measures, to the spread of exotic mosquito species.

In the study reported here, we describe the first detection of *Anopheles hyrcanus* in Poland. Mosquito sampling was carried out in fields designated for water infiltration (51°04′58.16″ N, 17°06′42.20″ E; Fig. 1) that are located on the outskirts of the southeastern part of the Polish city of Wrocław, along the left bank of the Odra River. Wrocław is one of the warmest cities in Poland and is characterized by a humid, temperate continental climate. The mean annual temperature in the region is approximately 9.0 °C, and the average annual precipitation is approximately 600 mm [10]. The entire region belongs to the Odra catchment area, where the Bystrzyca, Ślęza, Oława and Widawa rivers form an extensive system of ditches and tributaries. This aquatic ecosystem was constructed in 1896 to provide surface and underground water filtration of 160 m^3^ water/day for drinking water production. It was selected for this study because it is characterized by a high diversity of different aquatic and semi-aquatic habitats that provide ideal breeding sites for mosquitoes [11].

**Fig. 1:**
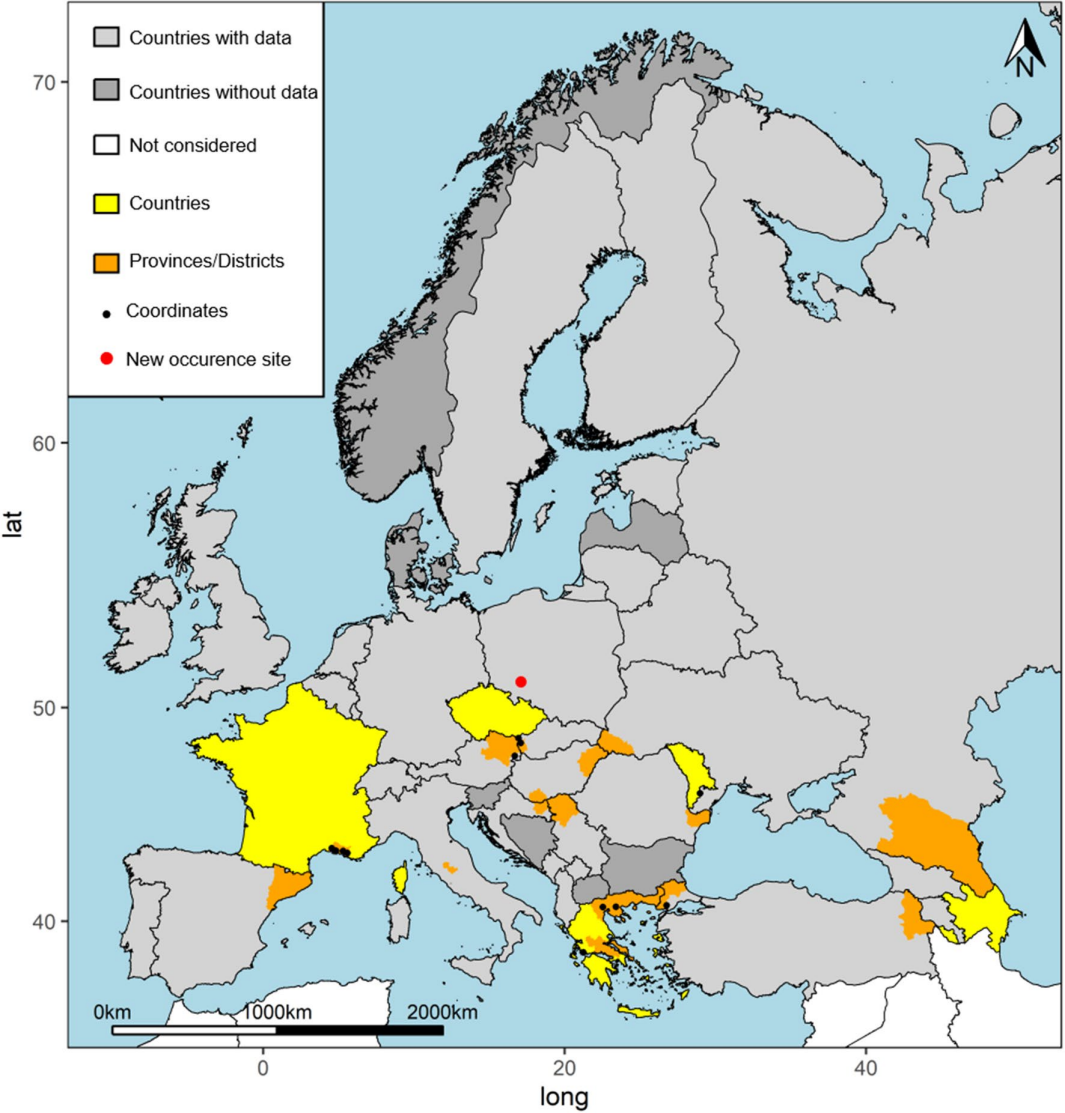
Occurrence map of *Anopheles hycranus* sensu lato at three different geographical levels. Countries are given in yellow, and provinces/districts are shown in orange. The filled black circles are global positioning system (GPS) coordinates derived by Bertola et al. [15]. The red dot indicates the new occurrence site reported in this study

The area is mostly marshy and dominated by water reservoirs, such as floodplains, wells and ponds, and by 12 km of canals and ditches. Willow-poplar alluvial forests dominated by white willow (*Salix alba*) and white poplar (*Populus alba*) are the predominant vegetation cover along the river banks. Swamp vegetation, including high rushes and large sedge communities with common reed (*Phragmites australis*), sweet flag (*Acorus calamus*), acute sedge (*Carex gracilis*), yellow iris (*Iris pseudacorus*) and common sedge (*Carex fusca*), are found near both stagnant and flowing waters. The high diversity of aquatic biotopes results in a high species diversity [12]. The area is contained within the sanitary protection zone and is protected under the Natura 2000 sites, a European network of protected nature areas, as hornbeam forest in the Odra River Valley; it is one of the 118 important areas in Poland that meet the criteria for Important Bird Areas. Thus, the area plays important roles in both nature conservation and the water supply of Wrocław, and also hosts a large mosquito population that shows almost yearly massive emergence [11].

Adult mosquito collections were conducted with CO_2_-baited Encephalitis Vector Survey (EVS) traps (BioQuip Products Inc., Rancho Dominiquez, CA, USA) at two locations along the Odra river: Starodworska (51°4′21.48″ N, 17°6′49.55″ E) and Świątnicka (51°4′54.33″ N, 17°5′46.54″ E). The traps were installed 1 m above the ground and run overnight approximately from 1600 hours to 800 hours the following day. In Starodwoska, four collections were performed in July and September 2019 and four collections in July, August and September 2020. In Świątnicka, two collections were conducted in July and August 2019 and 2020. Mosquitoes were transported in dry ice containers to the laboratory where they were sorted under a stereo microscope and identified to species level using the dichotomous keys described by Becker et al. [1].

In 2019 and 2020, a total of 11,194 female mosquitoes were collected in Starodworska and Świątnicka (Table 1). The most abundant species was *Aedes vexans* (86.8% of all collected specimens), followed by *Aedes sticticus* (7.5%) and *Anopheles maculipennis* sensu lato (1.2%). Less frequent species were *Culex pipiens* sensu stricto/*Culex torrentium* (0.7%), *Aedes cinereus/geminus* (0.2%), *Anopheles claviger*/*Anopheles petragnani* (0.2%), *Aedes annulipes* group (0.2%), *Aedes rossicus* (0.1%), *Aedes geniculatus* (0.1%), *Culiseta annulata* (0.1%) and *Anopheles plumbeus* (< 0.1%). A remaining 136 specimens were damaged and their identity could not be determined.

**Table 1:** Mosquito taxa recorded in the study area of Wrocław, Southwest Poland during the sampling period in 2019–2020 with the number of female specimens collected, their respective overall proportion and proportion of specimens per sampling site are indicated in brackets

Sampling site	Year	Date	*Aedes vexans*	*Aedes* *sticticus*	*Coquillettidia richiardii*	*Anopheles* *maculipennis* sensu lato	Not determined Culicidae	*Culex* *pipiens * sensu stricto/*Culex. torrentium*	*Aedes* c*inereus/Aedes geminus*	*Anopheles claviger/Anopheles petragnani*	*Aedes* *annulipes* group	*Aedes* *rossicus*	*Aedes* *genicultus*	*Culiseta* a*nnulata*	*Anopheles plumbeus*	*Anopheles hyrcanus*	Number of specimens
Starodworska	2019	11 July	808	8	33	22	6	11	–	7	2	–	–	2	–	2	901 (8.0%)
		13 July	677	6	29	45	20	10	–	–	8	–	–	2	–	4	801 (7.2%)
		26 August	639	–	–	4	–	3	–	4	–	–	–	1	–	1	652 (5.8%)
		20 September	34	–	–	–	–	–	1	2	–	–	–	–	–	3	40 (0.4)
	2020	22 July	3244	647	8	13	60	10	4	2	6	2	2	–	–	1	3999 (35.7%)
		05 August	1027	9	2	15	13	9	11	1	2	6	2	–	–	2	1099 (9.8%)
		03 September	197		–	–	–	4	4	–	–	2		–		5	212 (1.9%)
		15 September	948	10	–	–	11	2	5	–	–	2	4	–	1	18	1001 (8.9%)
Świątnicka	2019	22 July	1281	6	69	21		22	–	–	–	–	–	1	–	1	1401 (12.5%)
		26 August	417	7	4	10	20	2	–	4	–	–	–	–	–	3	467 (4.2%)
	2020	22 July	84	135	2	6	4	6	1	0	–	–	1	2	–	1	242 (2.2%)
		19 August	359	7	2	2	2	3	–		–	–	1		2	1	379 (3.4%)
Number (%) of specimens collected			9715 (86.8%)	835 (7.5%)	149 (1.3%)	138 (1.2%)	136 (1.2%)	82 (0.7%)	26 (0.2%)	20 (0.2%)	18 (0.2%)	12 (0.1%)	10 (0.1%)	8 (0.1%)	3 (0%)	42 (0.4%)	11,194 (100%)

A total of 42 (0.4%) *An. hyrcanus* specimens were collected over the 2-year collection period (Table 1). This species was identified based on different morphological features: (i) two pale spots on the apical half of the costal wing margin; (ii) distinctly swollen base of the fore femora; and (iii) a mostly dark tarsomere 4 of the hind leg that was pale at the apex. The wing veins were covered with dark and pale scales, forming contrasting spots. The antennae were dark brown, and the basal five to seven flagellomeres showed only a few white scales.

To confirm the morphological identification of *An. hyrcanus*, we performed molecular barcoding of the COI (cytochrome oxidase subunit I) gene region. Six specimens were individually deposited in 2-ml safe-lock tubes (Eppendorf, Hamburg, Germany), followed by the addition of approximately 20 pieces of 2.0-mm zirconia beads (BioSpecProducts, Bartlesville, OK, USA) and 500 µl of cell culture medium (high-glucose Dulbecco’s modified Eagle’s medium; Sigma-Aldrich, St. Louis, MO, USA) to each tube. The specimens were then homogenized in the Qiagen TissueLyser (Qiagen, Hilden, Germany) for 2 min at 30–50 Hz. DNA extraction was conducted using the QIAamp viral RNA mini kit according to the manufacturer's instructions (Qiagen). DNA elutions were used to amplify the COI gene region [13]. A comparison of the sequences of the six *An. hyrcanus* specimens with sequences deposited in GenBank showed a homology of 99.8–100% with available sequences of *An. hyrcanus* respectively *Anopheles pseudopictus.* All sequences were identical. In this study, we followed Ponçon et al. [14] in accepting that *An. hyrcanus* and *An. pseudopictus* probably belong to the same species, based on genetic analyses. One representative sequence has been submitted to GenBank (accession no. MZ093049).

This is the first record of *An. hyrcanus* in Poland. The detection of a total of 42 specimens during different sampling sessions conducted in 2019 and 2020 at two different sites (Table 1) indicates that the species is well-established at these locations. These newly described sites also represent the most northern occurrence of *An. hyrcanus* in Europe reported to date (Fig. 1). *Anopheles hyrcanus* has been previously documented in the Ukraine and Czech Republic, but no sightings have been reported in Germany, Belarus, Slovakia, Lithuania and Kaliningrad (Russia) [15]. The environmental conditions at both of our sampling sites resemble those of breeding sites reported for *An. hyrcanus*, which has a preference for large, stagnant water bodies with a rich aquatic vegetation, including reeds [1].

The interpretation of data on new and individual foci of a species at selected sites is difficult as the presence of that species could be driven by various factors, such as environmental change, including climate warming or intensified sampling. However, different studies indicate that *An. hyrcanus* is spreading in Europe, with new foci detected in Serbia, Slovakia, Czech Republic and Austria [16]. This mosquito species is expected to continue to spread further across Europe as the trend towards increasing annual temperatures continue.

*Anopheles hyrcanus* is a highly mammalophilic mosquito species, predominantly feeding on cattle and horses, but also on humans [17]. The species is considered an important vector of malaria in France [18]. In addition, a potential role as vector for *Dirofilaria immitis* or *D. repens* can be expected [19]. This is especially important in the light of the ongoing import of human malaria [20] and the ongoing circulation of *D. repens* in Poland [21]. Therefore, the ongoing spread of the species in Europe must be further monitored carefully.

## Data Availability

All data are available in the manuscript or open databases.
